# Effects of Plant-Derived Polyphenols on the Antioxidant Activity and Aroma of Sulfur-Dioxide-Free Red Wine

**DOI:** 10.3390/molecules28135255

**Published:** 2023-07-06

**Authors:** Yi Ma, Kangjie Yu, Xiaojiao Chen, Huixiang Wu, Xiongjun Xiao, Liming Xie, Ziyun Wei, Rong Xiong, Xun Zhou

**Affiliations:** 1 College of Bioengineering, Sichuan University of Science and Engineering, Yibin 644000, China; 321086002314@stu.suse.edu.cn (K.Y.); chenxjma@163.com (X.C.); 321086002311@stu.suse.edu.cn (X.X.); 321082302113@stu.suse.edu.cn (L.X.); 321086002308@stu.suse.edu.cn (Z.W.); 321086002430@stu.suse.edu.cn (R.X.); 2 Engineering Technology Research Center of Special Grain for Wine Making, Yibin 644000, China; 3 Department of Light Industry Textile Garment Testing, Guangdong Testing Institute of Product Quality Supervision, Guangzhou 510670, China; cce.whx@gzhu.edu.cn (H.W.); zhouxun@adzjy.onexmail.com (X.Z.)

**Keywords:** plant polyphenols, SO_2_ substitute, cabernet sauvignon

## Abstract

Significant efforts have been made in recent years to produce healthier wines, with the primary goal of reducing the use of sulfur dioxide (SO_2_), which poses health risks. This study aimed to assess the effectiveness of three plant-derived polyphenols (dihydromyricetin, resveratrol, and catechins) as alternatives to SO_2_ in wine. After a three-month aging process, the wines were evaluated using analytical techniques such as high-performance liquid chromatography, colorimetry, gas chromatography–olfactometry–mass spectrometry, as well as electronic nose and electronic tongue analyses, with the purpose to assess parameters including antioxidant activity, color, contents of volatile aroma compounds, and sensory characteristics. The results demonstrated various degrees of improvement in the antioxidant activity, aromatic intensity, and sensory characteristics of wines using polyphenols. Notably, dihydromyricetin (200 mg/L) exhibited the strongest antioxidant activity, with increases of 18.84%, 23.28%, and 20.87% in 2,2-diphenyl-1-picrylhydrazyl, 2,2’azino-bis(3-ethylbenzothiazoline-6-sulfonic acid), and ferric-ion-reducing antioxidant power assays, respectively. Resveratrol (200 mg/L) made the most significant contribution to volatile aroma compounds, with an 8.89% increase in the total content of alcohol esters. In E-nose analysis, catechins (200 mg/L) showed the highest response to aromatic compounds and the lowest response to volatile sulfur compounds, while also exhibiting the best sensory characteristics. Therefore, the three plant-derived polyphenols investigated here exhibited the potential to enhance wine quality as alternatives to SO_2_. However, it is important to consider the specific impact of different polyphenols on wine; hence, suitable antioxidants should be selected in wine production according to specific requirements.

## 1. Introduction

Sulfur dioxide (SO_2_) has become one of the most commonly used additives in the food industry, owing to its antimicrobial and antioxidant properties. Using SO_2_ as an additive in winemaking ensures antioxidant protection and microbial stability [1]. Despite these undeniable advantages, there are also certain health risks associated with the use of SO_2_. It has been reported that an ingestion of SO_2_ can cause adverse reactions in sulfite-sensitive wine consumers (approximately 1%), including headaches, diarrhea, skin rash, and bronchoconstriction [2]. Additionally, the observed bioaccumulative nature of SO_2_ is linked to the development of lung cancer [3]. For these reasons, its usage is strictly regulated by law [4], and current research aims to reduce or even eliminate the use of SO_2_ in the production chain through alternative control methods.

Several techniques have been employed to regulate the use of SO_2_ in the winemaking process, such as the application of high-pressure pulses [5], ultraviolet irradiation [6], and electric fields [7,8]. Although these methods have demonstrated potent antimicrobial properties, they have poor efficacy against free radicals, and their industrial applicability is limited. The use of chemical alternatives provides a more direct, effective, and cost-efficient approach; chemicals such as ascorbic acid, thiol compounds, chelating agents, and even natural products such as lysozyme and bacteriocins are being tested [9,10]. However, these alternatives are disadvantaged by an inadequate antioxidant capacity or may produce toxic gases, thereby affecting the wine quality [11]. Consequently, there is currently no compound or treatment method that can fully replace the use of sulfur dioxide in winemaking.

Polyphenols are bioactive compounds obtained from diverse plant sources, exhibiting potent antioxidant properties and serving as efficient scavengers of free radicals [12]. Recent reports indicate that phenolic compounds exhibit antibacterial activity through various mechanisms, such as hindering the formation of bacterial biofilms and cell walls (by inhibiting peptidoglycan), as well as preventing the depletion of bacterial enzymes and substrates [13]. According to the study of García-Ruiz et al. [14], polyphenols exhibit inhibitory effects on various lactic acid bacteria strains that affect wine fermentation, such as *Oenococcus oeni*, *Lactobacillus hilgardii*, and *Pediococcus pentosaceus*. Flavonoids and stilbenes exhibit the most potent inhibitory effect among the compounds under consideration. The findings indicate that phenolic compounds possess the capability to serve as a substitute for sulfur dioxide in the process of wine manufacturing. Undoubtedly, the wine industry has also taken notice of this phenomenon. In the production of Cabernet Sauvignon wine, Pastor et al. [15] employed resveratrol at concentrations of 150 and 300 mg/L in lieu of SO_2_. They discovered that the inclusion of resveratrol in Cabernet Sauvignon wine resulted in a more aesthetically pleasing color, without any discernible impact on its fundamental physical and chemical properties or sensory attributes. Irene et al. [16] employed grape stem extract, which is rich in polyphenols, to reduce the use of SO_2_ during the wine production process. Their findings indicated that the incorporation of grape stem extract in wine with reduced sulfur content resulted in an improved sensory quality and enhanced resistance to oxidation. Current research on polyphenols in wine primarily focuses on their role as supplementary agents or accessory pigments [17,18,19,20]. Although these studies demonstrate that polyphenols possess the ability to increase the ester content and enhance the quality of wine, it is important to note that their primary objective does not involve the reduction of SO_2_ use. Currently, there is insufficient empirical evidence supporting the use of polyphenols to replace SO_2_ in various applications.

At present, the focus of food and medicine research is directed toward dihydromyricetin (DMY), a flavonoid that is derived from *Ampelopsis grossedentata*. DMY has been shown to possess strong antioxidant properties, both in vitro and in vivo [21]. Dergacheva et al. [22] noted that DMY exhibited superior scavenging efficacy toward reactive oxygen species (ROS) compared to resveratrol, epicatechin, and other compounds. Previous research has confirmed the considerable inhibitory impact of DMY on food-borne pathogenic microorganisms, including *Vibrio haemolyticus* and *Staphylococcus aureus* [23]. The bactericidal activity of DMY is primarily attributed to its dual effect of cell membrane impairment and DNA binding [24]. At the same time, DMY exhibits anticancer, hypolipidemic, and hepatoprotective properties. It has been observed that the acceleration of the liver ethanol (EtOH) metabolism and the mitigation of EtOH-induced pathologies can serve as a safeguard against alcohol-induced hepatic injury [25]. This clearly demonstrates the potential of DMY as a wine preservative, offering an opportunity to enhance the stability and quality of wine. This may lead to significant advances in the investigation and manufacturing of healthier wine. Unfortunately, DMY has not yet been investigated in wine-related research.

In this study, three plant-derived polyphenols with potential application in winemaking, namely, dihydromyricetin, resveratrol, and tea polyphenols, were employed in the brewing of Cabernet Sauvignon wine. The study aimed to investigate changes in free radical scavenging capacity, color, volatile flavor substances, and sensory indexes of wines from various groups. In addition, we evaluated the feasibility of replacing SO_2_ with polyphenol in the process of wine production.

## 2. Results and Discussion

### 2.1. Physicochemical Analysis

Table 1 shows the general enological parameters of different wine samples before bottling. No significant differences were found in pH, soluble solids, total acidity, volatile acidity, alcohol content, and sugar content between the experimental group and the control group S. The results indicate that the addition of polyphenols at this particular concentration (150, 200 mg/L) had no negative impact on the alcohol metabolism of *Saccharomyces cerevisiae*. This is in agreement with the study of Diao et al. [26], who studied the use of plant polyphenols instead of SO_2_ in the production of Korla pear wine. The authors concluded that the different yeast growth trends during fermentation did not affect the physicochemical indexes of the produced pear wine. Some differences were observed between the physicochemical indexes of group CK and the other groups, because no antioxidants were added.

### 2.2. Analysis of Antioxidant Capacity

**Figure 1:**
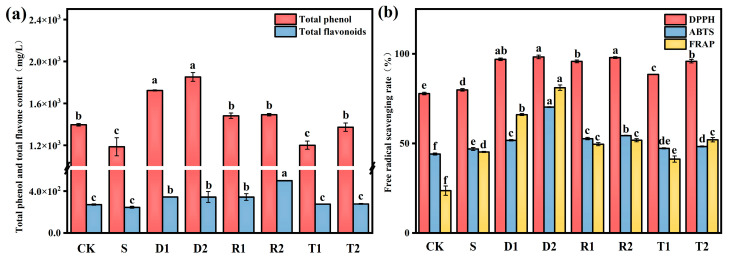
(**a**) Concentration of total phenolic and flavone (mg/L) quantified in grape varieties, analyzed under different conditions; Free radical clearance in grape varieties analyzed under different conditions; (**b**) Different superscript letters (a–f) for the same parameter denote significant differences (*p* < 0.05).

The results of the determination of the total phenol and flavonoid contents in wine are presented in Figure 1a. The experimental groups, with the exception of T1, exhibited a significant increase (*p* < 0.05) in total phenol content compared to control group S. The D1 and D2 groups exhibited the highest total phenol contents of 1622.46 and 1752.37 mg/L, representing significant increases relative to the control of 36.67% and 47.83%, respectively. This result can be attributed to the direct incorporation of exogenous polyphenols. Moreover, the addition of polyphenols resulted in an increased production of total phenols in wine. The impact of polyphenols on the adsorption capacity of yeast autolysate, pectinase activity, and *β*-glucosidase activity during fermentation affects the levels of free phenols [27]. Pastor et al. [15] found that different concentrations of resveratrol can promote the presence of other polyphenols in wine. Anaya et al. [18] also revealed a similar pattern for catechins, although no reports are currently available on the impact of DMY on the content of phenolic compounds in wine. The results of the present experiments indicate that DMY also has the ability to enhance the production of phenolic compounds in wine; moreover, the DMY-based enhancement of the total phenol content in wine is superior to that of resveratrol and catechins at the same concentrations. Dihydromyricetin and resveratrol are phenolic compounds belonging to the family of flavonoids. This is a significant contributing factor to the overall increase in the flavonoid content. Group R2 exhibited the highest concentration of flavonoids (498.35 mg/L). Furthermore, the present study revealed that SO_2_ had no significant impact on the overall concentrations of phenols and flavonoids (*p* < 0.05).

The antioxidant capacity of wine was assessed using three distinct free radical scavenging assays. The results shown in Figure 1b reveal that the wines in groups D and R, which were supplemented with flavonoid antioxidants, exhibited a higher antioxidant activity compared to those in group S. Although the free radical scavenging capabilities of wines containing tea polyphenols were lower than those of the other two polyphenols, the observed differences between wines with tea polyphenols and group S were not statistically significant (*p* < 0.05). These findings indicate that group D2 exhibited the highest activity for scavenging free radicals in all three tests, and thus possessed the highest antioxidant activity. As anticipated, the CK group exhibited the lowest antioxidant capacity in the absence of antioxidants. According to a previous report, phenolic compounds have the ability to extend the lifespan of yeast by mimicking the effect of Sir2 under calorie restriction conditions [28]. In addition, phenolic compounds have the potential to stimulate antioxidant enzymes, while suppressing the activity of enzymes responsible for generating ROS, namely lipase, NADH oxidase, and xanthine oxidase, thereby providing antioxidant protection. Dergacheva et al. [22] revealed that DMY exhibited the most significant inhibition effect on ROS and the strongest antioxidant effect among phenolic compounds, such as resveratrol and epigallocatechin. These findings are in agreement with the present experimental results.

**Figure 2:**
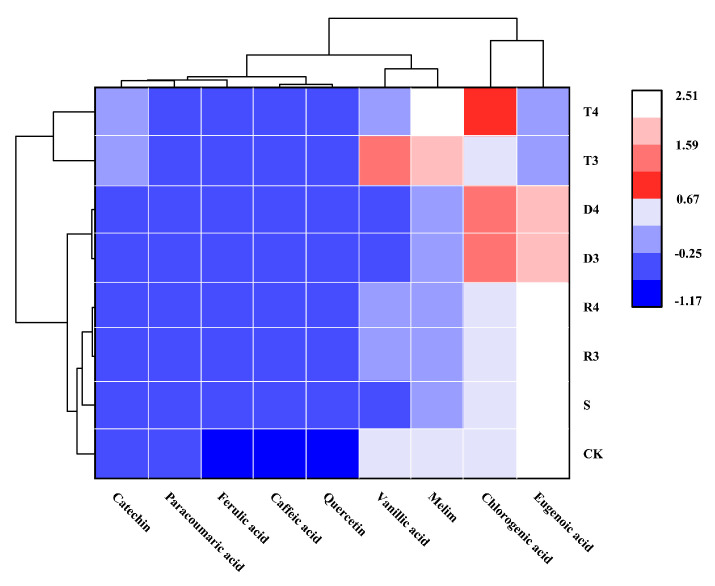
Concentration of phenolic composition (mg/L) quantified in grape varieties analyzed under different conditions.

A clustering heat map was used for processing and analyzing data, by visually comparing the variations in phenolic acid content among wines classified into different groups (as shown in Figure 2). Phenolic acid, a polyphenolic compound of considerable importance found in wine, displays antioxidant characteristics and plays a role in the nutritional and health benefits associated with wine consumption. In the present study, high-performance liquid chromatography (HPLC) was employed to quantitatively assess the presence of nine common phenolic acids in various types of wine. The results showed that various phenolic antioxidants had no statistically significant influence on the concentrations of coumarinic acid, ferulic acid, caffeic acid, and quercetin. Incorporating tea polyphenols has the potential to increase the levels of catechin, chlorogenic acid, rutin, and vanillic acid, while concurrently suppressing the production of syringic acid. We also observed that DMY inhibited the production of syringic acid and promoted the production of chlorogenic acid. The impact of resveratrol on the levels of phenolic acids was found to be negligible, with the exception of an increase in the production of vanilloid acid. Garaguso et al. [29] pointed out that the use of SO_2_ does not affect the phenolic acid content of wine. Castellari et al. [30] reported that, owing to the antioxidant properties of sulfur dioxide, SO_2_-free organic wine may be oxidized in the fermentation stage of the brewing process, resulting in a lower phenolic acid content compared with traditional wine. We observed that the ferulic acid, caffeic acid, and quercetin contents were significantly lower in the CK than the S group. These results are consistent with those of Castellari.

**Figure 3:**
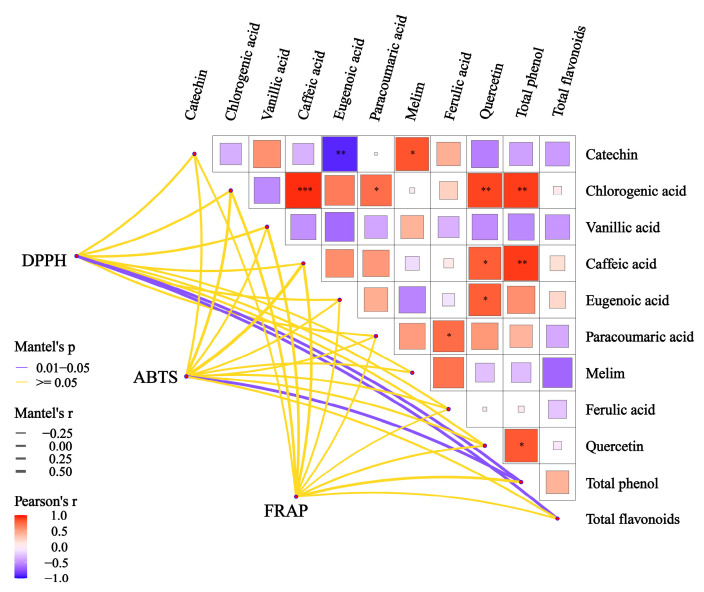
The thick line in the heat map of the correlation network of total phenols, total flavonoids, and phenolic acids was used to indicate the degree of correlation, and the color was used to indicate significance. In general, we did not think the correlation was statistically significant until *p* < 0.05. Results of a paired-sample *t*-test based on phenolic acid content. Red and blue asterisks denotepositive correlation and negative correlation, respectively (paired-sample *t*-test: *, *p* < 0.05; **, *p* < 0.01; ***, *p* < 0.001).

The correlation network heat map displayed in Figure 3 was employed to investigate the association between changes in the antioxidant properties of polyphenols and variations in the levels of total phenols and phenolic acids. The analysis revealed a statistically significant positive correlation (*r* > 0.5) between the total phenol content and the 1,1-diphenyl-2-picrylhydrazylradical (DPPH) and 2,2’-azinobis(3-ethylbenzthiazoline-6-sulphonate) (ABTS) scavenging abilities (*p* < 0.05). Additionally, a positive correlation (*r* > 0.5) was observed between the total flavonoid content and the ABTS scavenging ability (*p* < 0.05). Although phenolic acids had a favorable impact on the antioxidant activity of wine, they did not represent the main factor responsible for the changes in antioxidant activity observed in this study. Furthermore, the results were not statistically significant (*p* > 0.05). These findings suggest that the enhancement of the antioxidant capacity was not due to changes in individual or selected phenolic acids, but could be attributed to a qualitative shift resulting from the increase in the overall phenol content.

### 2.3. Analysis of Wine Color

Group S was employed as the control group to measure the luminance (L*), hue (a*), and hue b* values of the samples. Then, we calculated the color difference, de* (see below), and the results are presented in Table 2. The L* value of the wine samples in groups other than S exhibited a significant decrease following the aging process. However, the samples in group D2 did not display any statistically significant difference (*p* > 0.05) in this regard. This reflected the presence of polyphenol oxidase (PPO), peroxidase (POD), and other related enzymes in wine. The regulation of the phenolic compound biosynthesis and oxidation by PPO and POD can potentially facilitate the browning process and produce black, brown, and yellow hues [31]. The CK group exhibited the most obvious attenuation trend, presumably due to the absence of any antioxidant protection. The degree of redness of wine is determined by the positive value of the a* parameter. A higher value of a* was found to be positively correlated with a more intense and reddish–purple hue of the wine [32]. Wine treated with DMY had a greater a* value and a more pronounced red color compared to that in group S (*p* < 0.05). Wine made with other polyphenols was less valuable (*p* < 0.05) and had a more brownish–green appearance than that with SO_2_. A positive correlation was found between the b* value and the yellow hue of the wine [33]. Certain polyphenolic compounds exhibiting a yellow hue underwent a gradual dissolution process, leading to the formation of polymeric dyes with a yellow chromaticity [34]. This phenomenon ultimately resulted in the intensification of the yellow coloration. The results of our previous experiments indicate that the addition of polyphenols can facilitate the increase in the free polyphenol levels, which may be the primary factor contributing to the higher b* value in the experimental cohort.

**Figure 4:**
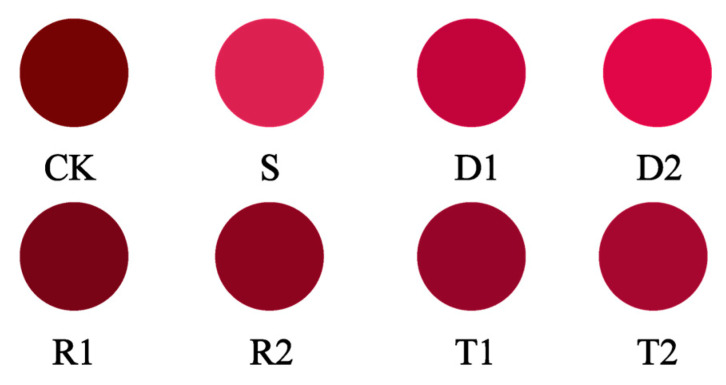
Characteristic color diagram of the wine samples.

The de* value represents the color difference between a sample and the standard, with higher values denoting a larger difference. The interaction between polyphenols and anthocyanins via molecular stacking, hydrogen bonding, and other mechanisms creates stable compounds that preserve the food color [35]. The experimental groups exhibited lower de* values compared with the CK group, and a reduced color difference relative to the S group. Furthermore, it was observed that in the concentration experimental design, an increase in polyphenol concentration resulted in a decrease in the de* value. The group supplemented with DMY (D2) exhibited the lowest de* value, and its chromatic difference with group S was minimal. The chromaticity diagram in Figure 4 reveals that, after a 3-month aging period, the wines in the S, D1, and D2 groups retained a violet–red hue, whereas those in the remaining groups exhibited a darker coloration, with a brown–black tone.

Oxygen dissolved in wine affects its ageing, and a small amount of oxidation can enhance the quality of red wine. SO_2_ can react with H_2_O_2_ to inhibit the Fenton reaction [36,37]. Phenols also can prevent Fenton reaction (reaction with oxygen or elimination of intermediate products in reaction links) from taking place [38,39]. According to Obradovic et al. [40], wine tannin (a polyphenol) can inhibit laccase activity, regulate dissolved oxygen, and prevent the color of wine from altering. Rasines-Perea et al. [41] indicate, for instance, that distinct phenols will result in various wine colors, which is related to the composition and source of polyphenols.

### 2.4. Analysis of Volatile Aroma Compounds

The selection of the D2, R2, and T2 groups from the three polyphenol experimental groups was based on their antioxidant activity and color, which were considered to reflect their brewing potential. The gas chromatography–olfactometry–mass spectrometry (GC–O–MS) technique was employed to identify the volatile compounds present in the samples. The retention index (RI) was used in conjunction with the mass spectra to characterize these compounds. A total of 34 aroma components were detected by both qualitative methods. A panel of four judges with expertise in GC–O analysis performed olfactory assessments on wine samples, characterized the volatile aroma compounds present in them, and recorded the corresponding flavor dilution factor (FD) values (Table 3). In instances where FD ≥ 64, it was deemed that the scent emanating from the sample was readily detectable and made a substantial contribution to the overall aroma characteristics of the wine. The analysis found that 18 aroma components satisfied the established criteria. Notably, phenylethyl alcohol, ethyl decanoate, ethyl octanoate, and ethyl acetate, which exhibited an FD value ≥ 512, were identified as the primary aroma components of wine. These compounds were also detected in other wine varieties, including Sauvignon Blanc, Semillon, and Gelatina [42]. The experimental group exhibited a significant increase in the FD values of 1-butanol and hexanol. The GC–O detection revealed that the above compounds exhibited a striking fruit aroma. Octanoic acid, (*R*,*R*)-2,3-butanediol, ethyl undecanoate, isoamyl decanoate, phenethyl propionate, methylhexyl ketone, and 2,4-di-*tert*-butylphenol were detected through both mass spectrometry and RI measurements. However, these compounds were not detected via GC–O in the present study.

The internal standard method was utilized to quantify the exact concentrations of 18 substances, consisting of 7 alcohols, 10 esters, and 1 acid, with FD ≥ 64. The concentrations and relative standard deviations of these compounds are presented in Table 4. Following the exogenous introduction of plant-derived polyphenols, only two volatile compounds, ethyl butyrate and ethyl nonanoate, exhibited no statistically significant variations.

With the exception of tea polyphenols, the remaining two polyphenols were found to have a significant impact on the alcohol content of wine, particularly in control group S, with the exception of 3-methylthiopropanol and group R2 propanol. Phenethyl alcohol and isoamyl alcohol were found to be the predominant alcohol compounds in wine; the same result was found in the experimental investigation conducted by Cheng et al. [43]. Phenylpropanol exhibited a distinctive floral scent and was the main aroma compound in Cabernet Sauvignon wine. Group R2 exhibited the highest phenylethanol content, resulting in the most intense floral odor [44]. The isoamyl alcohol compound was found to possess a cheese-like aroma, and was identified as a possible odorant that might have played a role in the study [45]. Interestingly, group R2 also exhibited the highest concentration of this compound. The incorporation of polyphenols sourced from plants showed the potential to substantially increase the concentration of primary alcohols in wine (*p* < 0.05), while also having a favorable impact on the overall olfactory profile of the wine. The observed results could be attributed to the impact of polyphenolic compounds on the enzymatic activities of decarboxylation or dehydrogenase within the Ehrlich pathway; this influences the metabolism of amino acids, specifically phenylalanine, methionine and tryptophan, by yeast, ultimately leading to an increase in alcohol content [44]. According to Ferreira et al. [46], higher alcohols (excluding phenylethanol) negatively contributed to the aroma of wine, particularly in high concentrations. Plant-derived polyphenols may result in elevated levels of higher alcohols in wine, which can result in a bitter taste and unpleasant aroma, potentially leading to headaches [47]. The 3-methylthiopropanol compound is characterized by a strong aroma reminiscent of scallions and meat, and has been observed to influence the sensory properties of wine. The inclusion of any test polyphenol resulted in a significant reduction in the concentration of 3-methylthiopropanol, with the lowest concentration observed in group T2 (24.87 μg/L); the FD value of 3-methylthiopropanol in the T2 group satisfied the criterion discussed above. The high concentration of 3-methylthiopropanol (41.45 μg/L) observed in group S can be attributed to the impact of SO_2_ stress on yeast, which facilitated the assimilation of sulfur and consequently led to an increased production of 3-methylthiopropanol, a derivative of methionine [48,49]. This phenomenon was not observed in the other groups.

Esters are known to have a favorable impact on the overall quality of wine, as they impart characteristic fruity aromas [50]. The ester concentrations exhibited significant variations across the five groups. Two main types of volatile esters are generated during the process of wine fermentation, namely, acetate and medium-chain fatty acid (MCFA) ethyl esters. Acetate is synthesized from acetic acid and ethanol under the catalytic action of acetyltransferase (encoded by ATF1 and ATF2) [51]. The incorporation of polyphenols resulted in a notable increase in the levels of acetate compounds, such as ethyl acetate, isoamyl acetate, and phenethyl acetate, compared to the S group (*p* < 0.05). The R2 group exhibited the most obvious effect. However, no obvious differences in FD values were found between different groups. Polyphenol can cause pyruvate and acetaldehyde to remain in yeast cells, thus enhancing the metabolic flux of acetyl-CoA [52]. This is related to the formation of esters. This conclusion was confirmed by the acetate contents recorded in this study. The variation in the concentration of MCFA esters exhibits a higher degree of complexity, as their production is influenced by various factors, including the wine matrix, yeast, external environment, and other related variables [53]. MCFA esters are formed through the reaction of ethanol and MCFAs catalyzed by two acyl-coenzyme A transferases, namely, ethanol *O*-acyltransferase encoded by EHT1 and EEB1 [51]. Ethyl esters have smaller molecular size and exhibit lipophilic properties, facilitating their diffusion from the cytoplasm to the extracellular medium. On the other hand, the long hydrocarbon tail of fatty acid esters reduces their ability to diffuse across membranes. Consequently, the impact of ethyl esters on the flavor and aroma of wine is more significant than that of MCFA esters [54]. The results of the present experiments are largely consistent with this pattern, as higher sensory descriptors (FD values) were observed for ethyl esters such as ethyl acetate and ethyl phenylacetate. However, the analysis also found that some esters such as ethyl caprylate and ethyl decanoate exhibited FD values above 512, indicating their substantial contribution to the aroma of wine. Furthermore, Liu et al. [55] obtained increased levels of unsaturated fatty acid (UFA) mixtures in wine by overexpressing the acyl-coenzyme A transferase encoded by EEB1. This led to an increase in ethyl caprylate and ethyl decanoate levels. Similar effects were observed with polyphenols. The quantitative analysis of volatile compounds showed an increase in ethyl caprylate and ethyl decanoate levels. This experiment revealed that different plant polyphenols had distinct effects on different esters. More specifically, the content of lauric acid ethyl ester decreased in the D2 group and increased in the R2 and T2 groups. On the other hand, the hexanoic acid ethyl ester levels were found to decrease in the T2 group and increase in the D2 and R2 groups.

**Figure 5:**
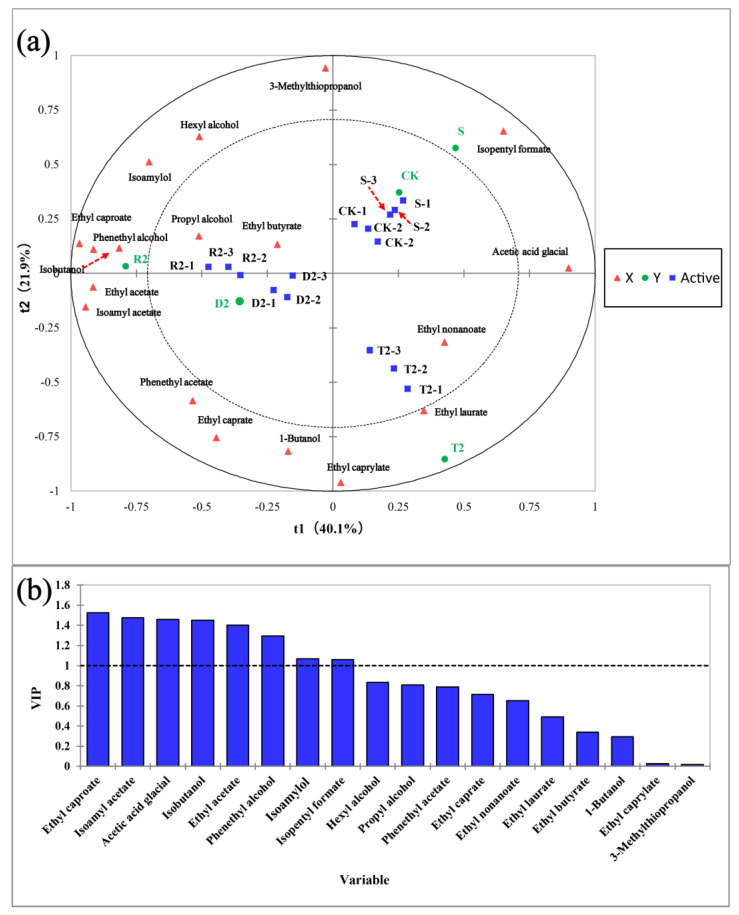
Partial least squares discriminant analysis (PLS-DA) of wine aroma among different groups. (**a**) Correlation plot of explanatory variable (X, volatile compound) and study category (Y, group) over the first two components (t1 and t2) of the PLS-DA model. Wine samples with activity representing CK, S, D2, R2, and T2. PLS-DA model shows the predictive power and overall fit [Q^2^ cum] = 0.813, [R^2^X cum] = 0.527, [R^2^Y cum] = 0.811; (**b**) Significance (VIP) score of explanatory variable (X, volatile compound) for prediction. Variables with VIP scores above 1.0 were considered important for prediction: ethyl caproate; isoamyl acetate; acetic acid glacial; isobutanol; ethyl acetate; phenethyl alcohol; isoamylol; isopentyl formate.

The partial least squares discriminant analysis (PLS-DA) method was employed to analyze the quantitative data of key aroma substances, with the aim to understand the differences among the various samples. The initial two constituents (t1 and t2) of the PLS-DA model effectively distinguished wines with different levels of antioxidants, which explained 62.0% of the total variance (proportion of explained variance in the *x* matrix (R^2^X) (cumulative) = 0.527; proportion of explained variance in the *y* matrix (R^2^Y) (cumulative) = 0.811; *Q*^2^ (cumulative) = 0.813) (Figure 5). Several compounds, including hexanol, isoamyl alcohol, ethyl decanoate, phenylethanol, isoamyl acetate, phenylethyl acetate, ethyl acetate, ethyl caproate, and *n*-butanol, exhibited a significant inverse relationship with t1. Wines that contain different antioxidants can be distinguished through t1. The CK group exhibited a greater degree of similarity to the S group, with higher concentrations of isoamyl formate and acetic acid. The introduction of acids altered the distinctive olfactory constituents of wine and increased the intricacy of its structure. Group D2 exhibited high concentrations of ethyl decanoate and *n*-butanol, resulting in a pronounced floral aroma of the wine. The T2 group exhibited high concentrations of ethyl nonanoate and ethyl laurate, which are known for their pronounced fruity aroma and refreshing leafy scent. Group R2 exhibited a correlation with several fragrance constituents, including phenylethanol, with the strongest scent. The VIP score, describing the influence of variables on the prediction, is illustrated in Figure 5. Overall, metabolites with a VIP value ≥1 are the primary discriminant factors among sample groups, and thus play a crucial role in the differentiation of samples from distinct groups. The figure shows that ethyl caproate was the dominant volatile compound, followed by isoamyl acetate, glacial acetic acid, isobutanol, ethyl acetate, phenethyl alcohol, isoamylol, and isopentyl formate. The introduction of polyphenols sourced from plants was found to alter the aroma profile of wine by modulating the levels of alcohol-derived ester aroma compounds.

### 2.5. Analysis of Electronic Tongue and Electronic Nose

**Figure 6:**
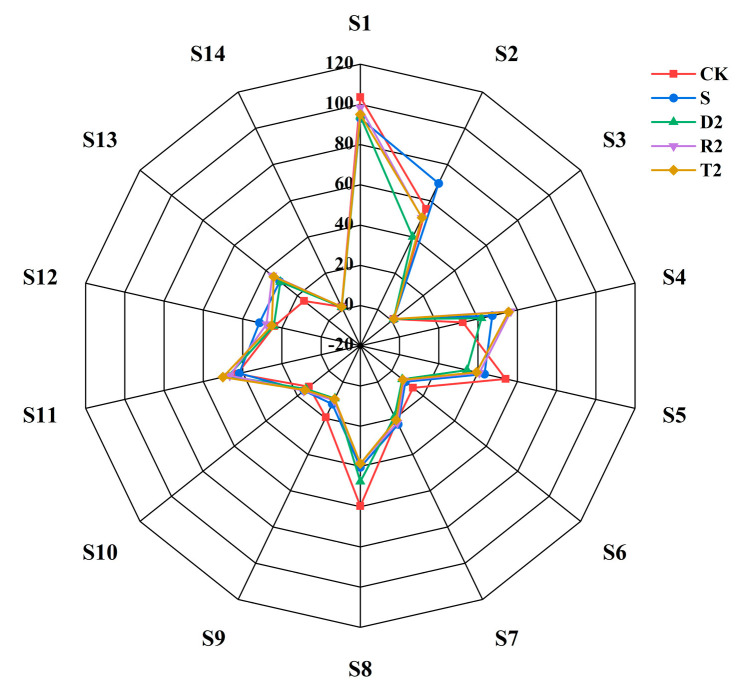
Electronic nose radar.

To investigate the sensory differences among different types of wine, we employed an electronic nose and an electronic tongue to analyze the specimens. Olfactory radar profiles for various wine samples were determined using the obtained sensor responses. The S2 (hydrogen sulfide) and S12 (sulfide) groups exhibited weaker responses in the absence of antioxidants or added plant-derived polyphenols compared to the S group (Figure 6). As shown in the analyses discussed in the previous sections, polyphenols derived from plants have the potential to reduce the levels of volatile mercaptans, especially for 3-methylthiopropanol, which could be associated with the reactions of the S2 and S12 probes. The S11 (aromatic compounds) probe exhibited the highest response within the T2 group and the second highest response within the D2 group. It was discovered in this investigation that group R2 had the strongest impact on the enhancement of the alcohol ester aroma in wine. Nonetheless, the aforementioned regulation did not reappear during the identification process carried out by the S11 probe.

**Figure 7:**
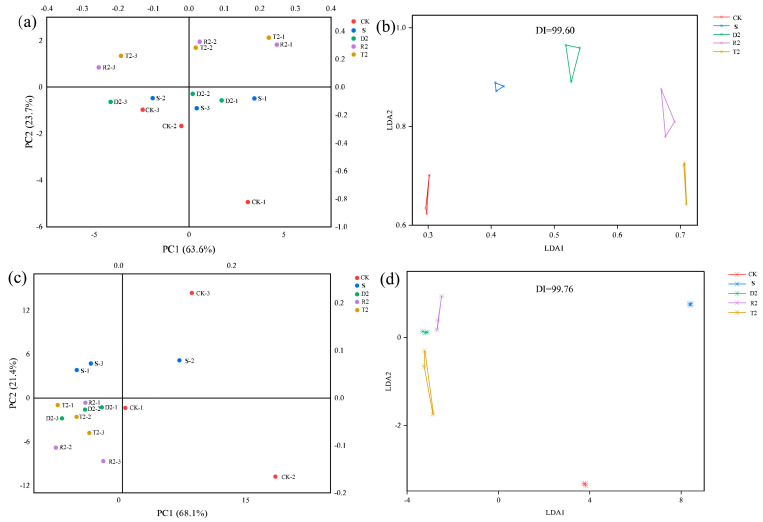
(**a**) Phenolic acid correlation network heat map PCA analysis of electronic nose; (**b**) Phenolic; (**c**) Phenolic acid correlation network heat map PCA analysis of electronic tongue; (**d**) Phenolic acid correlation network heat map DFA analysis of electronic tongue.

The results of the discriminant factor analysis (DFA) indicated that the discrimination index (DI) was 99.76 (Figure 7). The samples exhibited a minimal overlap, suggesting a significant difference in odor among the groups. This observation is consistent with the results of the PLS-D analysis presented in Figure 6. Principal component analysis (PCA) was conducted on the signal data of 14 probes to draw a two-dimensional diagram of the electronic nose response. Both group T and group R occupied two quadrants and exhibited a degree of similarity in their olfactory perception (Figure 7). The analysis suggests that the use of plant-derived polyphenols may enhance the production of alcohol esters, ultimately leading to an improved wine aroma. However, the PCA of group D2 highlighted a similarity to groups S and CK. These findings suggest that the olfactory perception of wine is not solely contingent upon the presence of specific volatile compounds; instead, there is a complex interplay among these compounds that synergistically contributes to the overall aroma profile of wine.

PCA and DFA analyses were employed to examine the e-tongue responses obtained from different wine groups. According to the results presented in Figure 7, the DI value was 99.60. The samples exhibited no significant overlap, indicating a notable difference in taste between the groups. The PCA results showed that groups D2, R2, and T2 exhibited a high similarity and occupied the same quadrant. Conversely, group S was located in the upper quadrant of the three plant-derived polyphenol groups, indicating a significant difference. Phenolic compounds have a significant impact on the overall quality of wine, and changes in the corresponding amounts can potentially impact the taste. Li et al. [20] investigated the sensory quality of red wine with tannin and concluded that the wine with hydrolyzed tannin received high ratings for aroma and bouquet, and that it would enhance the taste balance and have a pleasant aftertaste. Previous research suggests that excessive amounts of glycosides, catechins, and other compounds can contribute to the bitterness of wine [56].

## 3. Materials and Methods

### 3.1. Winemaking

Cabernet Sauvignon grapes were picked at the Shuanglong Winery in Huailai, China (114°28′–115°10′ E, 40°22′–41°03′ N) in September 2021. Huailai is one of the top-quality wine-producing areas in China. With an average elevation of 792 m, it is located in the semi-arid area of a middle temperate zone, belonging to the temperate continental monsoon climate with four distinct seasons, sufficient sunshine, and a large temperature difference between day and night. The wine picking was carried out under the guidance of wine brewing experts from the Sichuan Fruit Wine and Dew Wine Industry Research Institute. A total of 500 kg of grapes were picked by hand. Immediately after picking, they were delivered to the Engineering Technology Research Center of Special Grain for Wine Making, and subjected to de-embryonic and crushing procedures. The substance was transferred to a stainless steel tank with a capacity of 25 L. Pectinase was incorporated into the grape juice at a concentration of 0.03 g/L, followed by immersion at a low temperature of 4 °C for a duration of 2 days. Subsequently, white granulated sugar and citric acid were added in specific quantities to achieve a sugar degree of 22 °Brix and pH of 4.0, respectively. The process of alcoholic fermentation was initiated through the introduction of 0.2 g/L of commercially available *Saccharomyces cerevisiae* into a fermenter made of stainless steel. The complete process of brewing is executed at the brewery of the specialized Cereal Engineering Research Centre, where the alcohol and malolactic fermentation are conducted under regulated temperatures (25 °C).

In this study, oenological polyphenol additives, dihydromyricetin, resveratrol, and tea polyphenols were used. Polyphenol additives were selected, according to previous research [26,57]. Treatments with different concentrations of polyphenol supplementation are described below:

CK: control group with no treatment; S: the addition of 70 mg/L SO_2_;
D1: the addition of 150 mg/L DMY; D2: the addition of 200 mg/L DMY;
R1: the addition of 150 mg/L resveratrol; R2: the addition of 200 mg/L resveratrol;
T1: the addition of 150 mg/L tea polyphenols;
T2: the addition of 200 mg/L tea polyphenols.

Subsequently, the wine underwent a filtration process, was subsequently bottled, and subjected to storage under controlled temperature conditions (14 °C ± 1 °C) for a period of three months prior to analysis.

### 3.2. Standards, Gases, and Chemical Reagents

The polyphenol additive, primary aroma component standard, C7–C40 normal paraffin standard and phenolic acid standard utilized in this investigation were procured from PtSrTi (Chongqing, China).

The high-activity dry yeast utilized for fruit wine production was procured from Angel Yeast (Angel Yeast, Yichang, China). Chemical reagents such as Pectinase 500 U/mg, potassium metabisulfite, 3,5-dinitrosalicylic acid, phenol, methanol (HPLC), acetonitrile (HPLC), and formic acid (HPLC) were obtained from Shanghai Yuanye Biotechnology Co., Ltd. (Shanghai, China). Additionally, sodium bicarbonate, sodium potassium tartrate, DPPH (2,20-azinobis (3-ethylbenzothiazoline-6-sulfonic acid)) and glucose were acquired from Chengdu Kelong Chemical Reagent Factory (Chengdu., China).

The ABTS Assay Kit was procured from Suzhou Keming Biotechnology Co., Ltd. (Suzhou, China). The FRAP Assay Kit was procured from Suzhou Greis Biotechnology Co., Ltd. (Suzhou, China).

### 3.3. Analytical Methods

The wine samples were analyzed for key parameters, including alcohol strength (% *v*/*v*), reducing sugar, total and volatile acids, extracts, and pH values, using recommended procedures outlined in GB/T 15038-2006 [58].

Using T10 UV/Vis spectrophotometer (Beijing Puxi General Instrument Co., Ltd., Beijing, China), the free radical scavenging activity, total phenol and total flavonoid contents were measured. The Folin–Ciocalteu reagent measured the wine phenolic content [59]. A 10 mL graduated colorimetric tube included several gallic acid–wine dilution gradients. These solutions received 1.5 mL of 15% Na_2_CO_3_ and 1 mL of 0.2 mol/L FC reagent. Distilled water diluted the solution to 10 mL. The absorbance was measured at 765 nm after a one-hour dark incubation. Academically, the results are expressed in milligrams of gallic acid equivalent. The standard curve analysis shows that the absorbance (y) is related to gallic acid concentration (x) as y = 0.0012x − 0.0024. R^2^ = 0.9998.

The wine total flavonoid content was measured through a spectrophotometric assay [60]. These solutions were added to a 10 mL graduated colorimetric tube, and diluted with 95% ethanol to 5 mL. Next, 0.3 milliliters of a 5% sodium nitrite solution were added and thoroughly mixed. A 10% aluminum nitrate solution (0.3 mL) was inverted and incubated for 60 min. A blank control was made by mixing 2 mL of a 1.0 mol/L NaOH solution and 30% ethanol with the aluminum nitrate solution. The absorbance was measured at 510 nm via spectrophotometers. As per academic standards, the results are expressed in milligrams of a rutin equivalent. The standard curve analysis produced a linear relationship for absorbance and flavonoid content: y = 0.0061x + 0.0003. R^2^ = 0.9989.

Phenolic molecules have many antioxidant effects. Thus, the antioxidant capacity was determined using three different methods: ABTS radical scavenging assay, DPPH radical scavenging assay, and ferric ion reducing antioxidant power (FRAP). The DPPH assay was based on the method outlined by You et al. [61]. Dissolving 7.8864 mg of analytical purity DPPH in ethanol yielded a 100 mL solution. The DPPH solution and three milliliters of each red wine sample were mixed evenly. The mixture was incubated at 37 °C for 30 min in the dark. Absorbance was measured at 517 nm.

(1) $$\mathrm{DPPH}\;\mathrm{radical}\;\mathrm{scavenging}\;\mathrm{activity}\;(\%)=\frac{A\;\mathrm{control}-A\;\mathrm{sample}}{A\;\mathrm{control}}\times 100$$

“A sample” is the absorbance of the sample in the DPPH solution and “A control” is the absorbance of the DPPH solution in water.

The ABTS Assay Kit performed the radical scavenging assay according to the guidelines provided in the kit’s instructions. A working solution of 950 μL was combined with 50 μL of the wine sample and 50 μL of the extract to serve as the test and blank groups, respectively. The resulting mixture was incubated in the dark for 30 min, and the light absorption was subsequently measured at 734 nm to obtain the test solution.

(2) $$\mathrm{ABTS}\;\mathrm{radical}\;\mathrm{scavenging}\;\mathrm{activity}\;(\%)=\frac{A\;\mathrm{control}-A\;\mathrm{sample}}{A\;\mathrm{control}}\times 100$$

“A sample” is the absorbance of the sample in the ABTS solution and “A control” is the absorbance of the ABTS solution in PBS.

The FRAP Assay Kit measured FRAP according to the guidelines provided in the kit’s instructions. The experimental procedure involved the preparation of a blank group by mixing 150 μL of distilled water with 850 μL of color developing solution. The test group was prepared by mixing 75 μL of wine with 75 μL of distilled water and 850 μL of color developing solution. Both groups were incubated at 37 °C for 10 min in the absence of light. The measurement of absorbance was conducted at a wavelength of 590 nm.

(3) $$\mathrm{FRAP}\;\mathrm{radical}\;\mathrm{scavenging}\;\mathrm{activity}\;(\%)=\frac{A\;\mathrm{control}-A\;\mathrm{sample}}{A\;\mathrm{control}}\times 100$$

“A sample” is the absorbance of the sample in the FRAP solution and “A control” is the absorbance of the FRAP solution in water.

The quantification of phenolic acid was conducted through the employment of Shimadzu LC-2030 HPLC (Shimadzu, Japan). To make standard solutions, 50 mg of catechin, chlorogenic acid, vanillic acid, caffeic acid, eugenoic acid, paracoumaric acid, rutin, ferulic acid, and quercetin were diluted in 10 mL of ultra-pure water. Dilution and filtering via a 0.45 μm filter were performed on mixed composition standard solutions. ZORBAX SB-Aq, 4.6 mm × 250 mm, 5 μm particle size, was used in the experiment under 25 °C. A modified Del Pino-García et al. [62] method was used for chromatography. The experiment used mobile phases A and B. HPLC mobile phases A and B were 98% formic acid and 2% methanol, respectively. Time (t) in minutes and percentage B made up the gradient. The gradient scheme was (10, 30%), (35, 50%), (40, 60%), (45, 70%), (35, 70%), (45, 50%), (50, 5%), (55, 5%). The wavelength of the detector is 280 nm.

The method for determining the wine color was investigated in relation to Strati et al. [63]. The wine samples’ chromaticity properties were delineated by the chromaticity coordinates L* (photometric), a* (red/green), and b* (yellow/blue). The aforementioned data were acquired through the utilization of a CR-400 colorimeter of Tokyo, Japan origin, which underwent calibration via distilled water. The control group was designated as Group S. The assessment of the color parameters was conducted subsequent to a 90-day period of ageing. The mean value of the three measurements was obtained in a random manner from each specimen.

Chemical aroma standards were mixed at 50–2000 mg L^−1^. This covered all grape sample component concentrations. An 8-milliliter aliquot of wine was put into a 15-milliliter glass sample vial, preloaded with 1 g of NaCl and 20 microliters of a 2-octanol internal standard solution with a concentration of 0.458 mg mL^−1^. HS-SPME extracted volatile chemicals. PDMS fibers (Agilent Technologies, Palo Alto, CA, USA) and 45 °C for 35 min were used for optimum headspace SPME sampling. Following these conditions, the extracted compounds were injected into the GC-O-MS. The GC-O-MS investigation used an Agilent 8890 GC (Agilent Technologies, Palo Alto, CA, USA) with 5975C MS (Agilent Technologies, Palo Alto, CA, USA), and ODP4 olfactory detection port (GERSTEL, Shanghai, China). The improved CANUTI V technique quantified volatile organic molecules [64]. A DB-Wax column (60 m × 0.25 mm I.D., 0.25 μm film thickness) for the analysis. Helium carried the experiment at 1.2 mL min^−1^. Oven parameters: for 5.0 min, the experiment started at 40 °C. The temperature was raised to 60 °C at 2 °C min^−1^. A second temperature increase to 180 °C at 5 °C min^−1^ ensued. Finally, 230 °C was reached at 10 °C min^−1^. After 10 min at 230 °C, the oven returned to 40 °C. The cycle, including oven cool-down, lasted 58 min. In scan mode, the mass spectrometry detector covered 50–200 g. The mass spectrometry transfer line was always 240 °C. An electron bombardment ionization source (EI) at 230 °C and 70 eV was used in the experiment. Full scan acquisition and 150 °C MS quadrupole temperature was used. Methodology included a 3 min solvent delay. Injecting multiple n-alkanes simultaneously calculates the retention index. The interface temperature of the olfactory detector was 230 degrees Celsius, and the mixture ratio was 1:1. Four national tasters were trained for two weeks in a controlled testing environment in accordance with ISO 8589 (2010) specifications. Upon completion of the training, scent and describe the flavor substances, and record the FD value of the flavor substances using aroma extract dilution analysis.

E-nose (Shanghai Fenrui International Trade Co., Ltd., Shanghai, China) assessed the aroma profile’s similarity. E-nose analysis was developed from previous literature [65]. A glass vial held 20 mL of each wine sample. The sensor signal values reached equilibrium after 240 s of measurement. After measuring, the electronic nose program stored the data for PCA and LDA analysis. After each sample, the instrument’s alternative port flushed the sample gas channel with active carbon-filtered air for 120 s. Table 1 lists the 14 metal oxide sensors in the PEN 3.5 electronic olfactory instrument. Sensors react differently to volatile substances. The wine samples had three replicates. E-tongue (Shanghai Fenrui International Trade Co., Ltd., Shanghai, China) assessed the sample taste. E-tongue analysis was developed from previous literature [66]. A glass vial held 20 mL of each wine sample. At 1 hertz, the sample interval was 240 s. The sensors received a 60 s distilled water washing before and after each collection. The samples were collected three times. After measuring, the electronic tongue program recorded the data for PCA and LDA analysis (Table 5).

### 3.4. Statistical Analysis

All analyses were performed in triplicate. Data are expressed as the mean ± standard deviation (S.D.). In all cases, *p* ≤ 0.05 was considered to be statistically significant. All data processing was conducted using SPSS^®^ statistical software (IBM^®^ SPSS^®^ Statistics for Windows Version 25.0. IBM Corp, Armonk, NY, USA). Partial least-squares discriminate analysis (PLS-DA) was performed using XLSTAT 2023 statistical software (Addinsoft, Paris, France). The remaining graphs were created using Origin 2021 (Origin Lab, Northampton, MA, USA).

## 4. Conclusions

The contribution of SO_2_ to wine cannot be overlooked; however, adverse reactions to it affect the health-related properties of wine. Therefore, in order to produce healthier wines, it is necessary to explore the use of natural antioxidant additives to reduce or replace SO_2_. This study presents the first application of dihydromyricetin, a promising flavonoid in wine, with promising results. Dihydromyricetin (200 mg/L) exhibited the highest antioxidant capacity. Additionally, the present experiments confirmed that the contribution of polyphenols to the wine aroma and sensory quality resveratrol (200 mg/L) made the most significant contribution to volatile aroma compounds, with an 8.89% increase in the total content of alcohol esters. The electronic nose analysis revealed that catechins (200 mg/L) showed the highest response to aromatic compounds and the lowest response to volatile sulfur compounds, thus exhibiting the best sensory characteristics. Furthermore, the positive effects of SO_2_ during long-term aging should not be ignored. Hydrogen peroxide (H_2_O_2_) can trigger the Fenton reaction in wine, leading to the formation of hydroxyl radicals, which can affect the sensory properties of wine. In the presence of SO_2_, it reacts with H_2_O_2_ to reduce it to water. Compared to SO_2_, polyphenols can also eliminate quinone and H_2_O_2_ to maintain the wine dissolved oxygen balance. Long-term ageing of wine with various polyphenols results in distinct color characteristics. Consequently, the selection of suitable phenolic antioxidants may be contingent upon particular production requirements. Alongside that, extensive research has already demonstrated the positive effects of phenolic compounds in reducing oxidative stress, enhancing lipid metabolism, and regulating the gut microbiota. This is, of course, the next research focus for wines containing polyphenols as antioxidants.

## Figures and Tables

**Table 1:** Enological parameters of all wines.

Group	pH	Soluble Solids	Total Acidity (g/L) ^1^	Volatile Acidity (g/L) ^2^	Alcohol Content (%, *v*/*v*)	Sugar Content (g/L)
CK	3.49 ± 0.031 ^a^	7.53 ± 0.058 ^b^	9.19 ± 0.035 ^b^	0.56 ± 0.010 ^d^	10.43 ± 0.042 ^ab^	1.04 ± 0.010 ^a^
S	3.13 ± 0.015 ^b^	7.63 ± 0.15 ^a^	9.66 ± 0.034 ^a^	0.61 ± 0.026 ^bc^	10.41 ± 0.010 ^b^	1.03 ± 0.031 ^a^
D1	3.18 ± 0.010 ^b^	7.83 ± 0.21 ^a^	9.96 ± 0.514 ^a^	0.63 ± 0.012 ^bc^	10.45 ± 0.030 ^ab^	1.05 ± 0.030 ^a^
D2	3.17 ± 0.021 ^b^	7.87 ± 0.25 ^a^	9.72 ± 0.69 ^a^	0.64 ± 0.040 ^ab^	10.42 ± 0.015 ^ab^	1.03 ± 0.010 ^a^
R1	3.16 ± 0.068 ^b^	7.90 ± 0.20 ^a^	9.73 ± 0.22 ^a^	0.68 ± 0.040 ^a^	10.41 ± 0.011 ^ab^	1.04 ± 0.015 ^a^
R2	3.12 ± 0.025 ^b^	7.90 ± 0.26 ^a^	9.83 ± 0.32 ^a^	0.61 ± 0.012 ^bc^	10.45 ± 0.030 ^ab^	1.05 ± 0.0058 ^a^
T1	3.13 ± 0.026 ^b^	7.91 ± 0.12 ^a^	9.63 ± 0.16 ^a^	0.58 ± 0.020 ^cd^	10.46 ± 0.017 ^a^	1.09 ± 0.020 ^a^
T2	3.16 ± 0.026 ^b^	7.92 ± 0.11 ^a^	9.47 ± 0.35 ^a^	0.62 ± 0.010 ^bc^	10.45 ± 0.025 ^ab^	1.04 ± 0.046 ^a^

^1^ expressed as tartaric acid; ^2^ expressed as acetic acid. Different superscript letters (^a^, ^b^, ^c^ or ^d^) for the same parameter denote significant differences (*p* < 0.05).

**Table 2:** Color parameters of the wine samples treated before fermentation.

Group	L*	a*	b*	de* ^1^
S	43.12 ± 2.56 ^a^	60.54 ± 1.28 ^c^	11.33 ± 1.28 ^d^	--
CK	9.86 ± 1.28 ^f^	69.12 ± 2.56 ^a^	26.15 ± 1.11 ^a^	37.41
D1	33.91 ± 2.11 ^b^	65.32 ± 1.63 ^b^	15.11 ± 1.01 ^c^	11.04
D2	40.15 ± 1.21 ^a^	68.24 ± 1.45 ^ab^	18.23 ± 0.42 ^b^	10.76
R1	20.56 ± 2.56 ^e^	40.12 ± 0.21 ^e^	16.73 ± 1.42 ^bc^	30.9
R2	22.88 ± 2.45 ^e^	51.12 ± 2.12 ^d^	15.12 ± 1.65 ^c^	22.64
T1	25.12 ± 3.15 ^d^	53.45 ± 3.10 ^d^	12.15 ± 1.23 ^d^	19.36
T2	27.15 ± 1.21 ^d^	58.45 ± 2.10 ^c^	12.03 ± 1.25 ^d^	16.12

^1^ de* represented the color difference value, with Group S as the control, and the values were calculated based on the average values of a*, b* and L*. The calculation formula was as follows: de* = [(ΔL*)^2^ + (Δa*)^2^ + (Δb*)^2^]^1/2^. Different superscript letters (a–f) for the same parameter denote significant differences (*p* < 0.05).

**Table 3:** Qualitative analysis of volatile aroma components.

No.	CAS	Compound	RI ^1^	Odor Description ^2^	Basis of Identification ^3^	FD Factor ^4^				
						CK	S	D2	R2	T2
1	78-83-1	Isobutanol	1095	Solvent, Alcohol	RI, MS, O	128	128	128	128	128
2	1960/12/8	Phenethyl alcohol	1913	Honey, Lilac, Rose	RI, MS, O	512>	512>	512>	512>	512>
3	123-51-3	Isoamylol	1204	Cocoa, Floral, Malt	RI, MS, O	512>	512>	512>	512>	512>
4	67-63-0	Isopropanol	920	Floral	RI, MS, O	32	32	2	16	8
5	24347-58-8	(R,R)-2,3-Butanediol	1544	--	RI, MS	--	--	--	--	--
6	71-36-3	1-Butanol	1140	Fruity	RI, MS, O	64	16	32	128	128
7	111-27-3	Hexyl alcohol	1352	Banana, Flower,	RI, MS, O	64	64	64	128	64
8	505-10-2	3-Methylthiopropanol	1715	Smelly onion	RI, MS, O	128	128	128	128	64
9	71-23-8	Propyl alcohol	1061	Balsamic, Candy	RI, MS, O	128	128	128	128	128
10	123-25-1	Diethyl succinate	1673	Cotton, Fabric	RI, MS, O	4	4	4	4	4
11	627-90-7	Ethyl undecanoate	173	--	RI, MS	--	--	--	--	--
12	105-54-4	Ethyl butyrate	1056	Apple, Butter	RI, MS, O	512	512	512	512	512
13	106-33-2	Ethyl laurate	1842	Floral, Fruit, Leaf	RI, MS, O	1	--	2	--	1
14	628-97-7	Ethyl palmitate	2262	Wax	RI, MS, O	16	16	16	16	16
15	110-38-3	Ethyl caprate	1392	Apricot, Brandy	RI, MS, O	512>	512>	512>	512>	512>
16	123-29-5	Ethyl nonanoate	1545	Floral	RI, MS, O	512>	512>	512>	512>	512>
17	123-92-2	Isoamyl acetate	1065	Glue, Pear	RI, MS, O	256	256	256	256	256
18	2306-91-4	Isoamyl decanoate	1863	--	RI, MS	--	--	--	--	--
19	141-78-6	Ethyl acetate	892	Aromatic, Grape	RI, MS, O	512>	512>	512>	512>	512>
20	123-66-0	Ethyl caproate	1241	Fruity	RI, MS, O	512	512	512	512>	512
21	2035-99-6	Octanoic acid	1670	--	RI, MS	--	--	--	--	--
22	2198-61-0	Isopentyl hexanoate	1464	Anise, Fruit, Spice	RI, MS, O	8	8	8	8	8
23	106-32-1	Ethyl caprylate	1425	Fruity	RI, MS, O	512>	512>	512>	512>	512>
24	110-45-2	Isopentyl formate	1070	Apple	RI, MS, O	512	512	256	256	256
25	103-45-7	Phenethyl acetate	1275	Rose, Honey	RI, MS, O	512>	512>	512>	512>	512>
26	122-70-3	Propanoic acid, 2-phenylethyl ester	1351	--	RI, MS	--	--	--	--	--
27	112-05-0	Nonanoic acid	2170	Cheese, Sour	RI, MS, O	4	--	2	--	--
28	64-19-7	Acetic acid glacial	1434	Vinegar	RI, MS, O	64	128	64	64	64
29	124-07-2	Octanoic acid	2070	Cheese, Sour	RI, MS, O	8	16	4	4	--
30	105-57-7	Acetal	894	Creamy, Fruit	RI, MS, O	1	1	2	1	--
31	75-07-0	Acetaldehyde	744	Floral, Apple	RI, MS, O	32	32	32	32	32
32	100-52-7	Benzaldehyde	1508	Floral	RI, MS, O	4	2	4	2	--
33	111-13-7	2-Octanone	121	--	RI, MS	--	--	--	--	--

^1^ RI, retention index on the DB-WAX capillary column; ^2^ Odor description as perceived at the sniffing port during GC–O analysis. ^3^ Odorants were identified by comparing them with aroma standards based on the following criteria: retention index (RI), mass spectrometric detection (MS), and odor description (O); ^4^ The sample was subjected to aroma extract dilution analysis. FD refers to the dilution factor.

**Table 4:** Quantification of volatile aroma components.

Compound	Quantitative Ion (m/z)	Content (µg/L)					Slope	Intercept	R^2^
		CK	S	D2	R2	T2			
Isobutanol	43	225.68 ± 6.05d	244.80 ± 2.44c	270.23 ± 1.67b	292.47 ± 3.065a	228.056 ± 13.025d	18.429	−0.0267	0.9914
Phenethyl alcohol	91	2959.47 ± 38.013b	2400.81 ± 10.80c	2898.84 ± 51.74b	3136.40 ± 109.25a	2510.82 ± 101.74c	123.060	12.375	0.9844
Isoamylol	55	3854.48 ± 187.74b	4100.02 ± 101.01b	3943.321 ± 40.39b	4561.73 ± 366.52a	3476.42 ± 67.77c	28.998	−0.222	0.9915
1-Butanol	56	1.30 ± 0.26d	1.64 ± 0.31cd	2.27 ± 0.16c	5.49 ± 0.31b	7.053 ± 0.63a	18.146	0.288	0.9927
Hexyl alcohol	56	35.54 ± 0.96b	32.44 ± 0.98c	28.12 ± 0.48d	40.17 ± 1.54a	24.29 ± 1.77e	25.092	2.105	0.9914
3-Methylthiopropanol	106	34.56 ± 2.14b	41.45 ± 1.0020a	31.85 ± 1.22b	34.44 ± 0.97b	24.87 ± 3.58c	43.616	1.887	0.9971
Propyl alcohol	31	32.50 ± 2.17c	42.54 ± 1.13b	48.021 ± 2.23a	41.87 ± 1.010b	34.93 ± 1.84c	21.463	−1.976	0.9996
Ethyl butyrate	71	54.80 ± 2.47ab	55.78 ± 2.08ab	52.77 ± 2.52b	58.098 ± 0.61a	55.33 ± 0.48ab	69.456	13.700	0.9817
Ethyl laurate	88	64.75 ± 3.23bc	63.81 ± 1.48bc	60.56 ± 1.72c	80.13 ± 2.51b	135.039 ± 49.58a	32.348	0.974	0.9987
Ethyl caprate	88	374.02 ± 21.03c	278.97 ± 10.46d	478.21 ± 16.46a	412.66 ± 13.45b	451.53 ± 33.72a	526.260	23.624	0.9938
Ethyl nonanoate	88	106.92 ± 2.54a	106.12 ± 2.08a	98.45 ± 4.35b	106.12 ± 4.04a	112.063 ± 5.80a	435.160	56.432	0.9985
Isoamyl acetate	43	210.05 ± 4.41c	184.12 ± 4.72c	257.19 ± 40.84b	312.49 ± 8.25a	210.5577 ± 10.20c	95.243	13.743	0.9915
Ethyl acetate	43	268.02 ± 16.48bc	253.11 ± 16.17c	285.87 ± 4.38b	314.50 ± 16.49a	262.29 ± 7.70bc	32.459	2.532	0.9913
Ethyl caproate	88	142.67 ± 10.63c	131.62 ± 3.61cd	179.49 ± 18.58b	207.13 ± 7.64a	118.55 ± 1.73d	108.560	7.926	0.9813
Ethyl caprylate	88	381.67 ± 3.33c	385.85 ± 5.048c	449.99 ± 29.31b	419.18 ± 5.99b	508.22 ± 26.59a	311.780	74.783	0.9915
Isopentyl formate	55	3579.45 ± 68.72b	3756.59 ± 139.60a	3026.47 ± 44.87d	3170.13 ± 60.51cd	3237.10 ± 55.90c	90.197	17.662	0.9934
Phenethyl acetate	104	75.62 ± 0.77b	78.45 ± 0.99b	81.45 ± 1.10a	83.25 ± 1.71a	82.62 ± 2.64a	246.740	41.509	0.9943
Acetic acid glacial	43	35.47 ± 0.97c	50.72 ± 0.37a	32.85 ± 1.62c	22.78 ± 2.51d	44.87 ± 2.18b	8.698	−2.344	0.9935

Different small letters (a–e) for the same parameter denote significant differences (*p* < 0.05).

**Table 5:** Chemical sensors used in the electronic nose corresponding to different types of volatile substances.

Sensor Number	Sensor Name	Sensor Name
1	S1	Ammonia, amine
2	S2	H_2_S, sulfur
3	S3	Hydrogen
4	S4	Organic solvent (alcohol)
5	S5	Food cooking volatile gas
6	S6	Methane, biogas
7	S7	Flammable gas
8	S8	VOS
9	S9	Hydroxide, gasoline
10	S10	Alkane, flammable gas
11	S11	Aromatic compounds
12	S12	Sulfide
13	S13	Sterols, triterpenes
14	S14	Lactone, pyrazine

## Data Availability

All data available are presented in this manuscript.

## Sample Availability

No samples available.
